# Effectiveness of integrated interpretation of exome and corresponding transcriptome data for detecting splicing variants of genes associated with autosomal recessive disorders

**DOI:** 10.1016/j.ymgmr.2019.100531

**Published:** 2019-10-23

**Authors:** Mamiko Yamada, Hisato Suzuki, Yuichi Shiraishi, Kenjiro Kosaki

**Affiliations:** a Center for Medical Genetics, Keio University School of Medicine, Tokyo, Japan; b Section of Genome Analysis Platform, Center for Cancer Genomic and Advanced Therapeutics, National Cancer Center Research Institute, Tokyo, Japan

**Keywords:** Exome, Transcriptome, Alternative splicing, Mutation detection

## Abstract

**Purpose:**

Part of the weakness of exome analysis lies in the inability to detect aberrant splicing. An evaluation of the post-splicing mRNA sequence concurrently with genomic variants could improve the diagnostic rate. We aimed to investigate publicly available exome sequencing data and its matching transcriptomics data of phenotypically normal individuals to identify alternatively spliced variants from known genes associated with autosomal recessive disorders under the premise that some of the subjects could be carriers of such disorders.

**Methods:**

Aberrant splicing events and their triggering genomic variants were detected with the aid of Bayesian network method “SAVNet” which was originally developed for cancer genomics.

**Results:**

Forty aberrant splicing events including exon skipping, the creation of a new splice site, and the use of a cryptic splice site in response to the disruption of the authentic site were detected in 1916 genes among 31 of the 179 subjects from the 1000 Genomes Project. The predicted effects on proteins were either frameshift mutations (30) or large in-frame insertions or deletions (10). Five missense mutations and 2 silent mutations were reinterpreted as triggering major changes in transcript sequences. The detection rate of provisionally truncating pathogenic variants increased by 19%, compared with a conventional exome analysis.

**Conclusion:**

The coupling interpretation of exome and transcriptome data enhances the performance of conventional exome analyses through the proper interpretation of intronic variants that are outside of the GT/AG splicing consensus sequences and also allows the reinterpretation of “missense” or “silent” substitutions that can indeed have drastic effects on splicing.

## 1 Introduction

Exome sequencing has rapidly become the primary method for the discovery of new causative genes in rare diseases and for detecting causative variants in known disease-causative genes in patients with complex phenotypic features. Currently, the overall diagnostic rate of most clinical exome laboratories is limited to around 30% [1,2]. This relatively low diagnostic rate may reflect the restricted scope of exome analysis, which is largely limited to the detection of variants within the coding region. More specifically, current exome analysis covers only the coding sequences and intronic sequences flanking the exon-intron boundaries. Regarding intronic sequence variants, interpretation has been difficult apart from the GT/AG splicing consensus sequences [3]. Regarding coding sequence variants, the splice-disrupting potential should also be considered according to a recent in vitro mini-gene experiment, which showed that about 10% of exonic disease-associated alleles disrupt splicing [4]. Despite various efforts toward the development of in silico computer programs that can predict splicing abnormalities, the predictive performance has been less than optimal [3]. An evaluation of the post-splicing mRNA sequence concurrently with genomic variants could theoretically improve the diagnostic rate.

Whole transcriptome sequencing (RNA-Seq), is an effective method for the quantitative estimation of mRNA expression and for the qualitative detection of alternatively or abnormally spliced transcripts within cells, tissues, and organs where a pathogenetic process is in progress. Whole transcriptome sequencing has been successfully applied in several mutation analysis studies [5]. However, when only the quantitative and qualitative data obtained from whole transcriptome sequencing are considered independently from genome variants, a vast number of potential mutational events are not listed, and the detection of true causative mutation(s) has been a difficult task.

Coupling the interpretation of personal genomes (e.g., exome data) with their corresponding whole transcriptomes (e.g., RNA-seq data) provides a valuable clue to the detection of aberrantly spliced transcripts and their neighboring genomic variants that trigger abnormal splicing. Recently, Shiraishi et al. implemented an integrated analytic method of exome-whole transcriptomes data on a personalized level through the development of a novel algorithm/software known as “SAVNet: Splicing-Associated Variant detection by NETwork modeling” and have applied it effectively in the field of cancer genomics [6]. They evaluated aberrant splicing with the potential to cause variants present in cancer tissues but not in surrounding normal tissues.

**Fig. 1:**
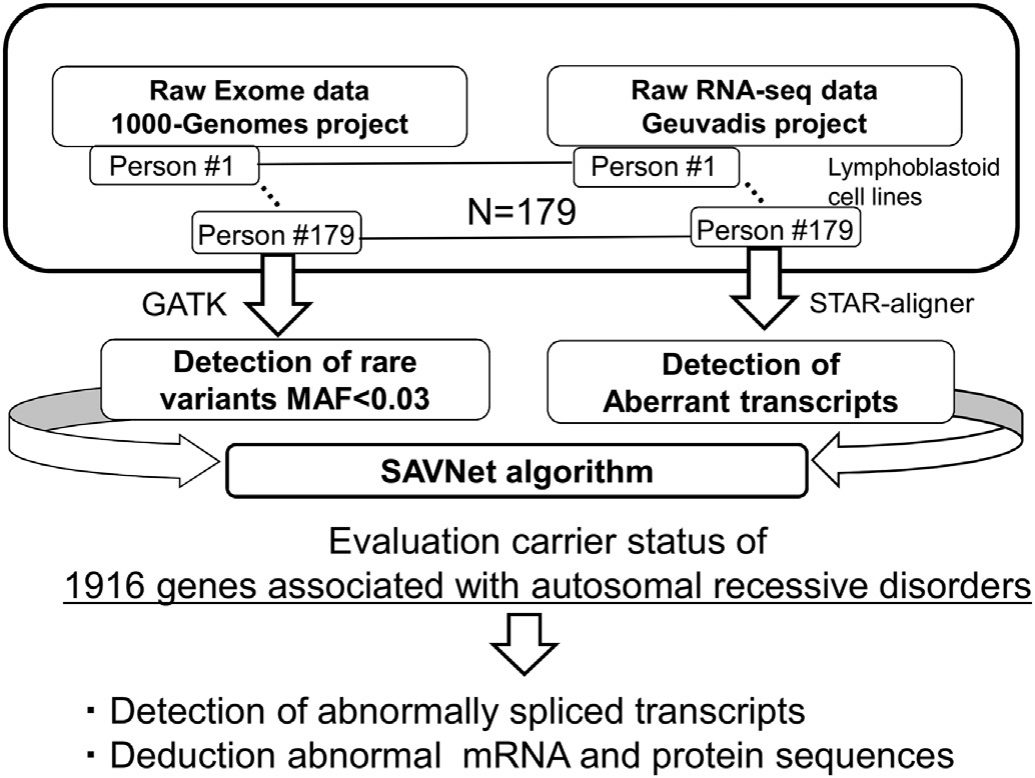
Overall flow of an integrated exome-transcriptome data analysis using the SAVNet algorithm. The 1916 known human genes associated with autosomal recessive disorders were screened for splicing-associated variants and their corresponding aberrant splicing among 179 phenotypically normal subjects who participated in both the 1000 Genomes and the Geuvadis projects.

In the present study, we aimed to evaluate how coupling the interpretation of personal genomes with their corresponding whole transcriptomes using the SAVNet algorithm could expand the current diagnostic limitations of an exome alone-approach in terms of detecting abnormal splicing in the genetic studies of rare diseases that are presumably caused by genomic variants that are rare in the general population. We aimed to investigate publicly available exome sequencing data and its matching RNA-Seq/transcriptomics data of phenotypically normal individuals [7] to identify alternatively spliced variants from a set of genes relevant to 1916 genes that are known to cause autosomal recessive disorders [8] under the premise that some of the subjects could be carriers of such disorders despite their normal phenotype (Fig. 1). We further aimed to evaluate the validity of the currently available in silico prediction methods for screening for genomic mutations that may cause aberrant splicing mutations.

## 2 Materials & methods

### 2.1 Materials

Publicly available raw exome and RNA-seq sequence data were investigated from the standpoint of splicing abnormalities as they relate to rare genomic variants (Fig. 1). We analyzed raw exome data from lymphoblastoid cell lines from phenotypically normal subjects recruited as part of the 1000 Genomes Project (www.internationalgenome.org) [7] and its associated transcriptomic project Geuvadis (http://www.internationalgenome.org/data-portal/data-collection/geuvadis) [9]. We evaluated samples from which both raw RNA-seq data and corresponding exome data were available on an individual basis. We restricted our analysis to a cohort of 179 (i.e., 87 subjects from Great Britain and 92 subjects from Finland) because of their relatively uniform genetic backgrounds.

### 2.2 Rare variant detection using exome analysis

For analyses involving individuals from the 1000 Genomes Project, hg19 aligned exome-sequence data in the bam format was downloaded from the 1000 Genomes Project website (http://www.1000genomes.org). The downloaded bam files were converted back to fastq files using the bam2fastq utility [10] so that we would be able to use our pipeline for mutation detection. Mapping of the sequence reads to the human reference genome (GRCh37) was performed according to the Burrows-Wheeler Aligner (BWA) [11] and the Genome Analysis Tool Kit (GATK) [12] best-practice guideline, as packaged in the integrated analysis suite variant tools [13]. Variant calls were accomplished by multiple callers including GATK [12], Isaac [14] and Scalpel [14,15]. The called variants were annotated with SnpEff [16]. Variants for which the minor allele frequency was less than 0.03 were fed into a SAVNet analysis as an initial list of genome variants that were possibly associated with splicing alterations for each gene.

### 2.3 Alignment of Geuvadis whole transcriptome data

Raw RNA sequencing data in the fastq format derived from lymphoblastoid cell lines originating from individual subjects who participated in the 1000 Genomes Project was downloaded from the European Nucleotide Archive (ERP001942). Genome indexes were generated using STAR version 2.5 [17] with the GRCh37 release 19 GTF file (ftp://ftp.sanger.ac.uk/pub/gencode/Gencode_human/release_19/gencode.v19.annotation.gtf.gz) and sjdbOverhang 100 option. Exon counts and gene counts were measured using BEDTools (intersectBed function) and HTSeq, which are implemented in the MAP-RSeq workflow [18].

### 2.4 SAVNet analysis

Splicing abnormalities were correlated with their corresponding rare variants using the.

SAVNet algorithm (https://github.com/friend1ws/SAVNet) [6]. The SAVNet algorithm consists of the following five steps: 1) the collection of evidence from different types of abnormal splicing, 2) the association of splicing alterations with rare variants to construct possible variant-splicing bipartite graphs, 3) the pruning of edges to select the best model explaining the data, 4) an evaluation of the false discovery rate by permutation, and 5) post-processing and rescuing splicing-associated variants (SAVs).

SAVNet identifies variants causing splicing alterations from among genome and transcriptome sequencing data focusing on the following two classes: 1) rare variants disrupting authentic splicing donor and acceptor motifs, with this class of variants leading to exon skipping, alternative 5′/3′ splice sites (SS) either within the exon or intron, or intron retentions; and 2) rare variants creating novel splicing motifs, with this class of variants leading to alternative 5′/3′ splice sites within either an exon or intron.

### 2.5 Reconstruction of transcript sequence derived from abnormal splicing

We restricted our analysis to 1913 genes that are known to cause autosomal recessive disorders [8] under the premise that some of the subjects could be carriers of such disorders even if they had been included in the 1000 Genomes Project cohort based on an apparently normal phenotype [7]. We confirmed that abnormal splicing event occurred when two or more abnormally spliced transcripts were present when aligned bam files of generated by STAR were visualized by Integrative Genomics Viewer (software.broadinstitute.org/software/igv/) [19]. First, a wild-type transcript sequence was downloaded from the RefSeq database [20]. Second, the wild-type transcript sequence was edited manually with the software Serial Cloner (http://serialbasics.free.fr/Serial_Cloner.html) and reconstructed so that the resultant sequence would incorporate abnormally spliced segment(s) that had been detected through the SAVNet analysis. We reconstructed the mRNA transcript sequences by knowledge based on junction Walk algorithm by Seok [21]. In short, we edited the ref-seq reference sequence to accommodate the alternative splicing event. Third, the reconstructed transcript sequence, which reflects the abnormal splicing, was translated into a hypothetical protein sequence. The deduced transcript/protein sequence was then compared with the wild-type transcript/protein sequence and the predicted mutational effects were expressed using the nomenclature by the Human Genome Variation Society.

### 2.6 Visualization of abnormal splicing events

Splicing events were visualized as a sashimi plot by drawing a connective element that illustrates the presence of a splice junction between two splice sites [22]. The information on read coverage along a gene was extracted from RNA-seq data and expressed as the sashimi-plot with curves connecting the splice sites [23,24].

### 2.7 Evaluation of deleteriousness in abnormally spliced variants

The deleteriousness of the abnormally spliced variants causing protein sequence indels was evaluated using the PROVEAN software (Protein Variation Effect Analyzer, http://provean.jcvi.org/) [25], which represents a region-based “delta alignment score” that measures the impact of an amino acid variation not only based on the amino acid residue at the position of interest, but also the quality of the sequence alignment derived from the neighborhood flanking sequences. PROVEAN is unique in that it is capable of evaluating amino acid insertions and deletions and multiple amino acid substitutions. PROVEAN scores of less than −2.5 were considered to be deleterious [25].

### 2.8 Evaluation of computer-aided prediction of abnormal splicing from genomic variants without transcript data

We tested whether the abnormal splicing detected using RNA-seq and the SAVNet software could be predicted by the prototypic software tools MaxEntScan (MES) [26] and SpliceSiteFinder-like (SSF) [27], both of which use current knowledge on the base composition of the splice site sequences and can predict aberrant splicing reasonably well [3]. The MES framework is based on the Maximum Entropy principle, whereas the SSF framework is based on the theory developed by Shapiro and Senapathy [27]. Both MES and SSF can predict whether an abnormal splicing could potentially occur, but they do not predict the resultant sequences of abnormally spliced transcripts. In the present study, abnormal splicing as demonstrated using a SAVNet analysis was considered to be the gold standard, and the predictions made by MES and SSF were evaluated as follows. First, the abnormal splicing predictions made by MES and SSF were considered “valid” or “successful” if the base change at the splicing-associated variant decreased the MES score by more than 15% and the SSF score by more than 5%, compared with the scores at authentic wildtype sites. Second, the MES-assisted prediction of the cryptic site use in the vicinity of the splicing associated variant was considered to be “valid” or “successful” if the MES score at the potential cryptic site was more than 80% of the MES score at the authentic wildtype splice site.

## 3 Results

### 3.1 Abnormal splicing events and their associated genomic variants

Among the 179 samples, the SAVNet program detected a total of 40 events of abnormal splicing and their associated variants within the known 1916 genes associated with autosomal recessive disorders (Table 1, Supplementary Table 1 for details).

**Fig. 2:**
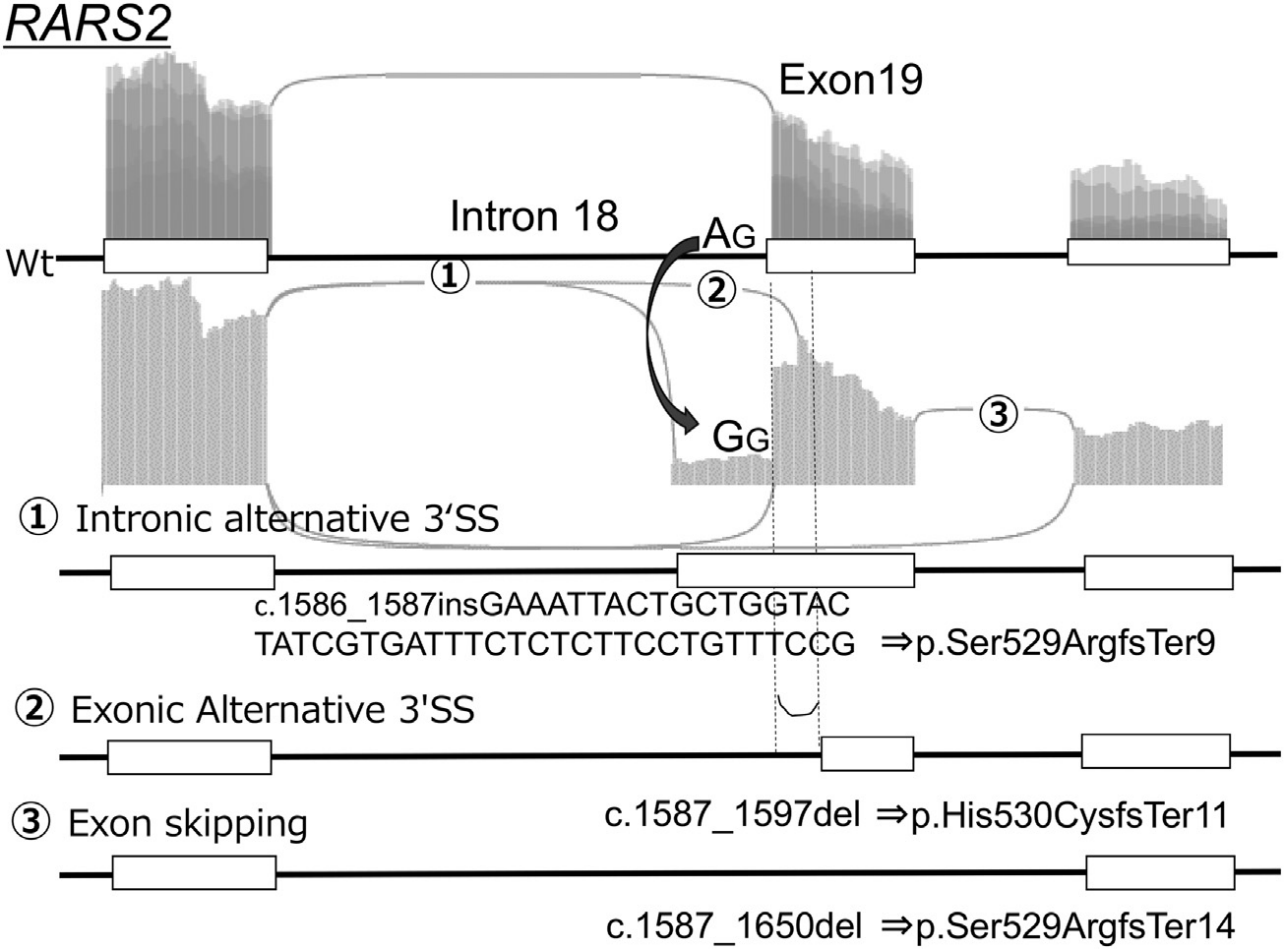
Multiple events of aberrant splicing were triggered by a single genomic variant (A > G) within exon 19 of the *RARS2* gene. The splicing-associated variant and its corresponding aberrant splicing are shown as a sashimi plot of the mutant allele. The boxes represent the exons, and the horizontal lines show the introns of the wildtype allele (WT) and three aberrant patterns (1–3). The splicing-associated variant A > G disrupted the authentic splice acceptor consensus dinucleotide “AG” within the 3′ end of intron 18. 1) The use of the cryptic splice acceptor site within intron 18, which was 5′ of the authentic acceptor site, resulted in a longer transcript that would lead to protein truncation because of a shift in the reading frame. 2) The use of the cryptic splice site within exon 19 resulted in a shorter transcript that would lead to protein truncation. 3) The skipping of exon 19 would lead to protein truncation.

All 40 abnormal splicing events detected by the SAVNet program, with the exception of *SUCLA2,* could be classified as follows according to the net effect on splicing and the location of the alternative splice sites, if any: exon skipping (*n* = 13; 6 disruptive events at the donor site and 7 disruptive ones at the acceptor site), exonic alternative 5′SS (*n* = 8), exonic alternative 3′SS (*n* = 10), intronic alternative 5′SS (*n* = 4), and intronic alternative 3′SS (n = 4). None of the SAVs were associated with intron retention. A representative example is illustrated in Fig. 2.

**Table 1:** Classification of splice-associated variants in view of the alteration of donors/acceptors.

	Donor					Acceptor				
	Exon skipping	Exonic alternative	Intronic alternative	Exonic alternative	Intronic alternative	Exon skipping	Exonic alternative	Intronic alternative	Exonic alternative	Intronic alternative
		5′ splice site	5′ splice site	3′ splice site	3′ splice site		5′ splice site	5′ splice site	3′ splice site	3′ splice site
Creation	None	*ACADVL*	*ADCY7*	None	None	None	None	None	*FOXRED1*	*NIN*
		*ERLIN2*	*CCT5*						*GLE1*^※3^	*RNASET2*
		*MUTYH*	*VARS2*						*GLE1*^※3^	
		*TCTN3*							*RUBCN*^※1^	
		*SNX14*^※1^								
Cryptic sites	*GBE1*^※2^	*GALNS*	*GBE1*^※2^	None	None	*GTSE1*	None	None	*NCAPD2*	*GUF1*
	*GYG1*	*GLMN*				*TRAPPC9*			*PAAF1*	*RARS2*^※4^
	*NDUFV1*	*CAD*^※1^				*LSS*			*RARS2*^※4^	
	*SP110*					*RARS2*			*ERCC8*^※1,5^	
	*NBAS*^※1^					*TCIRG1*			*ERCC8*^※1,5^	
	*TUBGCP3*^※1^					*KIAA0556*^※1^			*VPS13C*^※1^	
						*VPS53*^※1^				

Splice-associated variants were classified into three categories according to the mechanistic basis of the alterations at donor/acceptor sites. Columns 1 and 6 show “Exon skipping”, Row 1 of columns 2–5 and 7–10 show the “creation of a new splice site” that differs from the authentic splice site, and Row 2 of columns 2–5 and 7–10 show the “use of a cryptic splice site” because of the disruption of the authentic splice site caused by an SAV.

*1: Gene names with underlines represent splicing-associating variants annotated as ‘in-frame’. *2: Aberrant splicing patterns associated with two distinctive genomic variants in the same gene in separate individuals. *3: Aberrant splicing patterns associated with two slightly different genomic variants in the same gene in two separate individuals. The net effects of the two genomic variants at the transcript and protein levels were deduced to be identical. *4,5: Two distinctive aberrant splicing patterns associated with the same genomic variant in a single individual. *6: *SUCLA2*: excluded from this table. Highly complex pattern: pattern of two independent acceptor cryptic sites; exon skipping and creation of a completely novel exon within an authentic intron.

Generally, one SAV was associated with one aberrant splicing event and vice versa. The exceptions were as follows. For the *GBE1* gene, aberrant splicing patterns associated with two distinctive genomic variants of the same gene were observed in separate individuals. For the *GLE1* gene, aberrant splicing patterns associated with two slightly different genomic variants of the same gene were observed in two separate individuals. The net effect of the two genomic variants was deduced to be identical at the transcript and protein levels. In the *RARS2* and *ERCC8* genes, two distinctive aberrant splicing patterns were associated with the same genomic variant in a single individual, as discussed in the following subsection.

Aberrant splicing events were classified into three categories according to the mechanistic basis of the alterations at the donor/acceptor sites (Table 1): 1) exon skipping (Columns 1 and 6), 2) the creation of a new splice site that differed from the authentic splice site (Row 1 of columns 2–5 and 7–10), and 3) the use of a cryptic splice site because of a disruption at the authentic splice site because of an SAV (Row 2 of columns 2–5 and 7–10). Exon skipping resulted from either the disruption of the donor sites (*GBE1*, *GYG1*, *NBAS*, *NDUFV1*, *SP110*, and *TUBGCP3*) or the disruption of the acceptor sites (*GTSE1*, *KIAA0556*, *LSS*, *RARS2*, *TCIRG1*, *TRAPPC9*, and *VPS53*). Both the disruption of the donor sites and that of the acceptor sites tended to be associated with exon skipping events (i.e., 6/9 at donor sites and 7/13 at acceptor sites).

Eight donor creation events were observed. Among these 8 events, more events (i.e., 5 events: *ACADVL*, *ERLIN2*, *MUTYH*, *SNX14*, and *TCTN3*) occurred within the authentic coding sequence than within the intron authentic sequence (i.e., 3 events). Six acceptor creation events were observed. Among these 6 events, 4 events (*FOXRED1, RUBCN*, and two cases of *GLE1*) occurred within the coding sequence. As expected, alternative 3′ and 5′ SS were associated solely with the disruption of acceptor and donor sites, respectively. *SUCLA2* had a highly complex pattern and was not classifiable according to the categorization outlined above. The SAV NC_000013.10:g.48563118T > G variant led to an abnormal transcript that reflected two independent events: exon skipping of exon 3, and the creation of a completely novel exon within authentic intron 2.

The 40 abnormal splicing events were also classified into truncating or in-frame alterations based on the effects on the reading frame as deduced from the post-spliced mRNA sequences (Table 1). Truncating events were more common (i.e., 29/39, 74%). The SAVNet program detected 11 non-truncating alterations resulting from the use of an alternative splice site or exon skipping. These in-frame deletions or insertions were likely to be deleterious because of their large size, spanning from 1 amino acid residue to 44 residues and involving an average of 17 residues. All eleven in-frame deletions or insertions were predicted to be highly deleterious as per the PROVEAN program, with significant scores that were much lower than the threshold values.

### 3.2 Multiple abnormal splicing events triggered by one genomic variant

Two of the SAVs, one variant in *RARS2* and the other in *ERCC8*, were associated with more than one type of abnormal splicing. The NC_000006.11:g.88224740T > C variant of *RARS2* led to a more complex pattern of events: the disruption of the authentic acceptor sites led to the creation of an intronic alternative 3′SS, an intronic 3′SS, and exon skipping (Fig. 2). These three *RARS2* variants were observed in transcripts: c.1586_1587insGAAATTACTGCTGGTACTATCGTGATTTCTCTCTTCCTGTTTCCG, c.1587_1597del, and c. 1587_1650del (all were NM_020320.3). The variants resulted in protein sequence changes: p.(Ser529ArgfsTer9), p.(His530CysfsTer11), and p.(Ser529ArgfsTer14) (all proteins were NP_064716.2).

The NC_000005.9:g.60195555C > A variant in *ERCC8* led to the creation of two distinct acceptor sites (data not shown). Two *ERCC8* variants were observed in transcripts: one was c.618_629del, and the other was c.618_626del (both were NM_000082.3). The variants resulted in protein sequence changes: p.(Ser206_Ser209del) and p.(Ser206_Ser208del) (both were NP_000073.1).

### 3.3 Significant effect on reading frame triggered by single nucleotide variants within coding region

The interpreted effects of the SAVs on the reading frame needed to be adjusted in 7 samples once the RNA-seq information had been newly added to the preexisting information obtained from the exome analysis alone (Table 2): five abnormal splicing events in *ACADVL*, *ERLIN2*, *MUTYH*, *NIN*, and *TCTN3* had been interpreted as missense mutations when only the genome sequences were interpreted and annotated (SnpEff [16]). However, when information on abnormal splicing was taken into account, all 5 SAVs were identified as frame-disrupting mutations arising from aberrant splicing. Moreover, an apparently “silent” mutation in *NDUFV1* led to the disruption of the reading frame again because of the induction of aberrant splicing. Another apparently “silent” mutation in *SNX14* led to an in-frame protein sequence deletion of 12 amino acids. Numerical impact of non-canonical splicing abnormalities in comparison with putative loss-of-function variants was detected by exome analysis alone.

**Table 2:** Changes in interpretation of mutational consequences of splicing-associated variants depending on availability of information on aberrant splicing.

Gene (Cell line number)	Splicing-associated variants on genome coordinate system (GRCh37)	cDNA and protein sequence alteration deduced only from exome analysis		Aberrant splicing pattern unraveled by concurrent transcriptome analysis	Protein sequence alteration deduced from aberrant splicing pattern
Initially annotated as “silent” mutations					
*NDUFV1*	NC_000011.9:g.67379040G > A	NM_007103.3	NP_009034.2	Exon skipping	p.(Gly305AspfsTer12)
(HG00142)		c.1080G > A	p.(Ser360=)		(Frameshift change)
*SNX14*	NC_000006.11:g.86256864G > A	NM_153816.3	NP_722523.1	Exonic alternative 5' splice site	p.(Gly358_Val369del)
(HG00242)		c.1074C > T	p.(Gly358=)		(Deletion of 12 amino acids)

Initially annotated as “missense” mutations					
*ACADVL*	NC_000017.10:g.7127017A > G	NM_000018.3	NP_000009.1	Exonic alternative 5' splice site	p.(Glu412ArgfsTer9)
(HG00185)		c.1237A > G	p.(Ile413Val)		(Frameshift change)
*ERLIN2*	NC_000008.10:g.37607917A > G	NM_007175.6	NP_009106.1(LRG_1040p2)	Exonic alternative 5' splice site	p.(Glu187Ter)
(HG00242)		c.562A > G	p.(Ser188Gly)		(Nonsense change)
*MUTYH*	NC_000001.10:g.45796913C > T	NM_001128425.1	NP_001121897.1	Exonic alternative 5' splice site	p.(Ala473PhefsTer38)
(HG00256)		c.1417G > A	p.(Ala473Thr)		(Frameshift change)
*NIN*	NC_000014.8:g.51206145A > T	NM_020921.3	NP_065972.3	Intronic alternative 3' splice site	p.(Val1877HisfsTer3)
(HG00236)		c.5509 T > A	p.(Ser1837Thr)		(Frameshift change)
*TCTN3*	NC_000010.10:g.97442422G > A	NM_015631.5	NP_056446.4	Exonic alternative 5' splice site	p.(His480LeufsTer2)
(HG00120)		c.1438C > T	p.(His480Tyr)		(Frameshift change)

Top: Two SAVs (*NDUFV1* and *SNX14*) initially annotated as silent mutations were reconstructed as variants with a frameshift and a deletion, respectively, based on the transcriptome results.

Bottom: Five SAVs (*ACADVL*, *ERLIN2*, *MUTYH*, *NIN and TCTN3*) initially annotated as missense mutations were reconstructed as variants with a frameshift or a nonsense mutation based on the transcriptome results.

An analysis of the expression levels of each gene using MAP-RSeq showed that 1272 of the 1913 currently known causative genes of autosomal recessive disorders had exons with average read counts of 5 or more in the aligned RNA-seq data among the 179 samples. Regarding these 1272 genes, 38 nonsense mutations, 64 frameshift mutations, and 42 mutations involving the GT/AG splicing consensus sequences were identified by the exome analysis alone (data not shown). As shown above, an integrated analysis of the exomes and their matched transcriptomes detected 28 splicing-associated variants outside of the GT/AG splicing consensus sequences. Hence, an integrated analysis, rather than an exome-only analysis, increased the number of putative loss-of-function variants from 144 (38 + 64 + 42) to 172 (38 + 64 + 42 + 28), or 19%.

### 3.4 Reliability of computer-aided prediction of abnormal splicing from genomic variants without transcript data

We evaluated the performance of the MES [26] and SSF [27] software tools for predicting abnormal splicing events demonstrated through a SAVNet analysis. First, we considered the predictive performance of MES and SSF regarding 16 abnormal splicing events triggered by the disruption of the GT/AG splicing consensus sequences. The MES predicted a score of zero (i.e., complete abolition) for 15 of the 16 SAVs spanning the GT/AG splicing consensus sequences. At the remaining SAV, the MES and SSF software predicted a significant reduction in the score (i.e., more than 15% reduction using MES and more than 5% reduction using SSF). Hence, when Houdayer's criteria [3] were applied, MES and SSF correctly predicted the abnormal splicing for almost all the SAVs (i.e., 15/16) involving the GT/AG splicing consensus sequences. Among the 16 SAVs, 5 SAVs led to exon skipping, whereas 11 SAVs led to the use of a cryptic splice site. The MES-assisted predictions for 6 (55%) of the 11 SAVs that led to the use of cryptic splice sites were considered valid.

Second, we evaluated 14 SAVs that occurred in non-canonical donor/acceptor splice sites according to Cartegni [28]. The non-canonical donor/acceptor splice sites were defined as follows: 11 bases for the 5′ site (from the 3 last exonic bases to the 8 first intronic bases) and 14 bases for the 3′ site (from the 12 last intronic bases to the first 2 exonic bases). Among those 14 non-canonical donor/acceptor splice sites, 11 SAVs were within introns and 3 SAVs were within exons. MES and SSF correctly predicted the abnormal splicing in 12 of the 14 SAVs (86%) at the non-canonical donor/acceptor splice sites. Among these 14 SAVs, 7 SAVs led to exon skipping and 7 SAVs led to the use of cryptic splice site. The MES-assisted predictions for the 2 (29%) of the 7 SAVs that led to the use of cryptic splice sites were considered valid.

## 4 Discussion

An integrated analysis of exomes and their matched transcriptomes successfully detected rare genomic variants associated with abnormal splicing using the SAVNet algorithm, which was originally developed for cancer genome research [6]. An analysis of 179 phenotypically normal subjects detected 40 genomic variants associated with abnormal splicing in the known 1916 genes associated with autosomal recessive disorders.

Twenty-nine out of 40 of these variants (i.e., 73%) led to the disruption of the reading frame, and the remaining 11 variants caused in-frame insertions or deletions that were likely to be pathogenic because of their size.

A typical exome analysis kit covers not only the exonic coding regions, but also their flanking intronic regions for up to 20 or 30 bases. Nonetheless, the functional significance of such intronic variants usually remains unknown in conventional exome analyses, except for those occurring in GT/AG splicing consensus sequences. An integrated analysis with SAVNet enabled the direct evaluation of the transcriptional consequences of such non-canonical variants, identifying 16 variants among 179 subjects.

Moreover, an integrated analysis allowed the detection of abnormal splicing triggered by variants within the exonic coding sequences that had been annotated as non-synonymous variants or even as synonymous variants in a conventional exome analysis. Our findings regarding the importance of seemingly missense or silent mutations in triggering splicing abnormalities are compatible with recently reported experimental findings by Soemedi et al. [4], who used artificially designed mini-gene experiments to examine the splicing effect of “missense” or “silent” variants that had been determined to be “pathogenic” based on clinical grounds [4]. The experiments reported by Soemedi [4] were based on mini-genes with exons carrying the variants that were being studied along with their flanking exons and intervening introns, whereas the presently reported study observed naturally occurring events in lymphoblastoid cell lines in which all the exons of the transcriptional system were functioning in an intact manner. Our observations, which can be considered “prospective” rather than “retrospective” as in Soemedi et al.'s study [4], further recapitulate the importance of the splice-altering potentials of missense and silent mutations.

The detection efficiency of pathogenic alleles was significantly enhanced by coupling the interpretation of personal exome sequencing with the corresponding whole transcriptome. The number of SAVs detected through the presently reported study (40) was significantly large, compared with the total number (102) of nonsense variants and frameshift variants. One could argue that 12 of the 40 SAVs were mutations at canonical splice sites and therefore would have been detected by an exome analysis without relying on transcriptome data. Nonetheless, 28 of the 40 splicing mutations would not have been detected as pathogenic variants without the transcriptome analysis. Overall, we expect that the application of SAVNet analyses of lymphoblastoid cell lines in addition to standard exome analyses will increase the efficiency at which pathogenic alleles can be detected by about 20%.

Prototypic splicing prediction software (i.e., MES [26] plus SSF [27]) correctly predicted abnormal splicing in 12 of the 14 SAVs (86%) at non-canonical donor/acceptor splice sites. However, the MES-assisted prediction of cryptic site usage was only successful for a fraction of these sites (i.e., 29%). This observation indicates the incomplete capacity of such splice prediction software. The accumulation of data on splicing-associated variants, as documented in the present study, would eventually improve the accuracy of splice prediction software.

In the present study, SAVNet was applied to exome data combined with RNA-seq data originating from lymphoblastoid cell lines. Theoretically, a SAVNet analysis could be applied to an integrated exome-transcriptomic analysis of any type of organ tissue as long as the transcripts of interest are expressed at a measurable level. Thus, our observations could be extrapolated to organs including skeletal muscle, myocardium, kidney and liver, which are often investigated in biopsies, if the results are expected to be clinically useful. When organs for which a biopsy is not feasible are affected (e.g., the central nervous system), organoid tissues could be generated through the application of regenerative technologies, such blood-induced iPS cells, and these tissues could then be used to perform a combined RNA-seq and exome study [29].

Reassuringly, two thirds of all the genes (i.e., 1272/1916 genes) had expression levels that were amenable to a SAVNet analysis using mRNA from lymphoblastoid cell lines. Hence, even if mRNA is not available from the affected tissues or from induced tissues, a significant number of genes can be evaluated. The generation of lymphoblastoid cell lines from patient samples is routinely performed in research settings, but not in clinical settings. In view of the strong association between the expression profiles of lymphoblastoid cell lines and that in peripheral blood [30], when mRNA from globin mRNAs are removed using column purification [31], a SAVNet analysis of whole blood could represent a promising approach to clinical diagnostics.

The successful application of an integrated exome-transcriptome analysis that can effectively increase the sensitivity of mutation analyses has therapeutic significance because patients with some classes of splicing abnormalities can potentially be treated using splicing-modulating chemicals such as RECTAS [32], a rectifier of aberrant splicing that rectifies aberrant IKBKAP splicing in patients with dysautonomia, and TG003 [33], an inhibitor of CDC2-like kinase 1 (CLK1) that is a potential drug for Duchenne muscular dystrophy. The present study clarified precise splicing abnormalities in 40 lymphoblastoid cell lines. The availability of these 40 cell lines will facilitate the discovery and development of splicing modulating chemicals. When these splicing modulating chemicals become clinically available, patients who could be treated by such chemicals will need to be identified through a splicing analysis, such as a SAVNet analysis.

In summary, an integrated analysis that couples the interpretation of personal genomes with their corresponding whole transcriptomes using the SAVNet algorithm successfully detected how genome mutations affect transcripts and identified rare genomic variants associated with abnormal splicing. The application of integrated exome-transcriptome analyses will enhance the sensitivity of conventional exome analyses through the proper interpretation of intronic variants that are outside of the GT/AG splicing consensus sequences and even allow the reinterpretation of “missense” or “silent” substitutions, which can have drastic effects on splicing. Many of the inborn errors of metabolism are autosomal recessive disorders. Successful detection of pathogenic splicing mutations in the recessive genes, as illustrated in the present study, demonstrates that integrated interpretation of the exome and corresponding transcriptome data is a promising approach to significantly enhance the sensitivity of detection of the genetic causes in patients with inborn errors of metabolism. Last but not least, the precise definition of abnormal splicing could be of therapeutic significance in view of the rapid development of splicing-modulating agents.

The following are the supplementary data related to this article.

Supplementary Table 1 Forty events of abnormal splicing and their associated variants within the 1916 genes associated with autosomal recessive disorders. Data on allele frequency were derived from the gnomAD database (https://gnomad.broadinstitute.org). Allele frequency data on samples from individuals originated from Great Britain and Finland were derived from non-Finnish European and Finnish population, respectively. Supplementary Table 1

## Author contributions

M.Yamada and K.Kosaki designed the study. M.Yamada, H.Suzuki, and K.Kosaki analyzed the data. M.Yamada, H.Suzuki, Y.Shiraishi and K.Kosaki wrote the paper. All authors approved the final version of the paper.

## Disclosure

The authors declare no conflicts of interest.
